# Zao Ren An Shen capsule for chronic insomnia: Study protocol for a randomized, placebo-controlled trial

**DOI:** 10.1097/MD.0000000000014853

**Published:** 2019-04-05

**Authors:** Yoann Birling, Alan Bensoussan, Jerome Sarris, Nicole Avard, Xiaoshu Zhu

**Affiliations:** a NICM Health Research Institute, Western Sydney University, Westmead, NSW; b Professional Unit, The Melbourne Clinic, Department of Psychiatry, University of Melbourne, Melbourne, VIC; c School of Science and Health, NICM Health Research Institute, Western Sydney University, Penrith, NSW, Australia.

**Keywords:** Chinese Herbal Medicine, clinical trial, complementary and alternative medicine, insomnia, Zao Ren An Shen

## Abstract

**Background:**

Zao Ren An Shen (ZRAS), a Chinese Herbal Medicine product, has been proposed as an alternative to recommended treatments for chronic insomnia. There is a lack of strong evidence supporting this proposition.

**Aims:**

To assess the efficacy and safety of ZRAS capsule for chronic insomnia compared to placebo.

**Methods:**

A parallel-group, double-blind, randomized-controlled trial will be performed in Western Sydney University, Australia. After a 1-week placebo run-in, adults with chronic insomnia (n = 90) will be randomized in a 1:1 ratio to receive either ZRAS capsule or placebo for 4 weeks. Insomnia severity (Insomnia Severity Scale score), sleep parameters (measured with the Consensus Sleep Diary and actigraphy), fatigue levels (Fatigue Severity Scale score), psychological status (Depression Anxiety Stress Scale score), quality of life (Assessment of Quality of Life score), and adverse events will be assessed at baseline, mid-treatment, post-treatment and at a 1-month follow-up.

**Expected outcomes:**

We hypothesize that ZRAS capsule will improve insomnia severity, sleep parameters, fatigue levels, psychological status, and quality of life better than placebo at mid-treatment, post-treatment, and follow-up. We also hypothesize that the number of adverse events provoked by ZRAS capsule will be similar to placebo at these time-points.

**Trial registration:**

Australia New-Zealand Clinical Trial Registry (Registration number ACTRN12619000140156).

## 1 Introduction

Insomnia disorder is a chronic dissatisfaction with sleep quantity or quality resulting in significant distress or daytime impairment.^[1,2]^ With a prevalence rate of 7.9% to 15% in the general population,^[3–5]^ insomnia disorder is the most common sleep disorder. Long regarded as a symptom of other disorders, insomnia has been found to be a risk factor for the development of depression,^[6]^ anxiety disorders,^[7]^ chronic pain,^[8]^ hypertension,^[9]^ cardiovascular disease,^[10]^ leading to an impaired health-related quality of life,^[11]^ and a higher mortality risk.^[12]^ Because of insomnia-related absenteeism, decreased productivity, and health care expenses, insomnia is also a burden for the society, with a cost evaluated at $5010 per person per year in Canada.^[13]^

Pharmacotherapy and psychotherapy are the only treatments recommended by current guidelines.^[14–17]^ Despite being the most common medication for insomnia,^[18]^ Benzodiazepine Receptor Agonists (BzRAs) have been found to induce a large range of adverse effects,^[15,19]^ have been associated with rebound insomnia and withdrawal symptoms,^[20]^ and there are concerns about dependence.^[21,22]^ Unsurprisingly, insomniacs have a preference for nonpharmacological approaches.^[23,24]^ Cognitive Behavioral Therapy for Insomnia (CBT-I), a set of psychotherapeutic interventions used specifically to treat insomnia disorder, has been proposed as an alternative. The efficacy of CBT-I for insomnia disorder is well established^[20]^; however, the lack of trained practitioners prevents most of insomnia patients to benefits from this therapy.^[25]^

As recommended treatments suffer from the above drawbacks, many insomnia patients resort to over-the-counter medication, Complementary and Alternative Medicine (CAM) and self-help methods.^[26–28]^ Chinese Herbal Medicine (CHM) is one of the most common treatments for insomnia in Chinese populations ^[29,30]^ and its use is increasing in Western countries.^[31]^ Recent systematic reviews of randomized-controlled trials (RCTs) suggest that CHM is relatively safe and can effectively improve sleep quality and sleep parameters.^[32,33]^ However, the need of evidence for individual formulas was highlighted.^[32]^

Zao Ren An Shen (ZRAS) is a CHM formula composed of 3 herbs, that is, Suan Zao Ren (Ziziphi Spinosae Semen), Wu Wei Zi (Schisandrae Chinensis Fructus) and Dan shen (Salviae Miltiorrhizae Radix et Rhizoma), which have all sedative effects and low toxicity.^[34–41]^ This formula, manufactured in the forms of capsule, granule and solution, is indicated for insomnia.^[42]^ Clinical trials on ZRAS have showed a better effect than placebo and a similar or better effect than BzRAs.^[43–57]^ Adverse events were also less reported by participant taking ZRAS compared to BzRAs.^[43,44,47–51,53,54,56,57]^ However, none of these studies used appropriate methods of randomization and blinding. There is a need for high-quality RCTs.

The objective of this study is to assess the efficacy and the safety of Zao Ren An Shen capsule compared to placebo for chronic insomnia. As ZRAS is proposed as an alternative treatment and not a first-line treatment, we will use placebo as a comparator with a superiority framework. The primary outcomes are insomnia severity measured with the Insomnia Severity Index (ISI) for efficacy and the number of adverse events (AEs) for safety. The primary endpoint is post-treatment (week 4). We hypothesize that ZRAS capsule will show a better efficacy than and a similar safety to placebo. Secondary outcomes include objective and subjective sleep parameters, fatigue levels, psychological status, quality of life, adherence, and acceptability. The secondary endpoints are mid-treatment (week 2) and follow-up (week 8).

## 2 Methods

### 2.1 Study design

This study will use a randomized, double-blind, placebo-controlled design with a 1:1 allocation ratio and parallel groups. After 1 week of single-blinded placebo run-in, the participants will be randomized to the experimental group or the control group and take Zao Ren An Shen (ZRAS) capsule or a placebo, for 4 weeks. Outcomes will be assessed at pretreatment, mid-treatment, post-treatment, and at a 1-month follow-up (Tables 1 and 2).

**Table 1:**
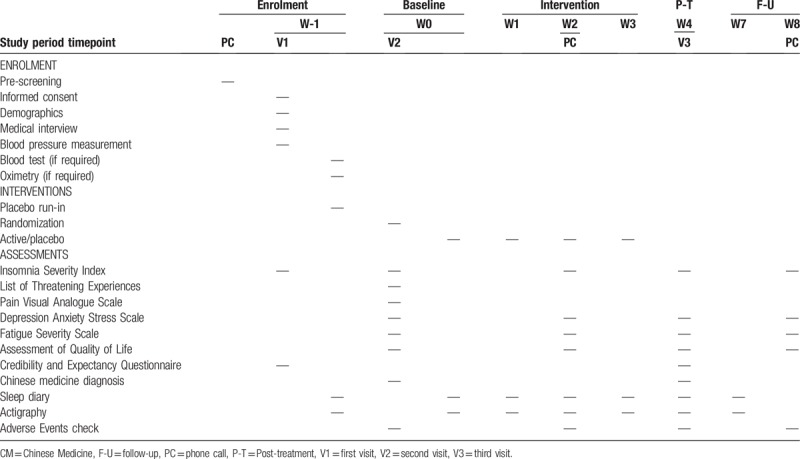
Trial procedures.

**Table 2:**
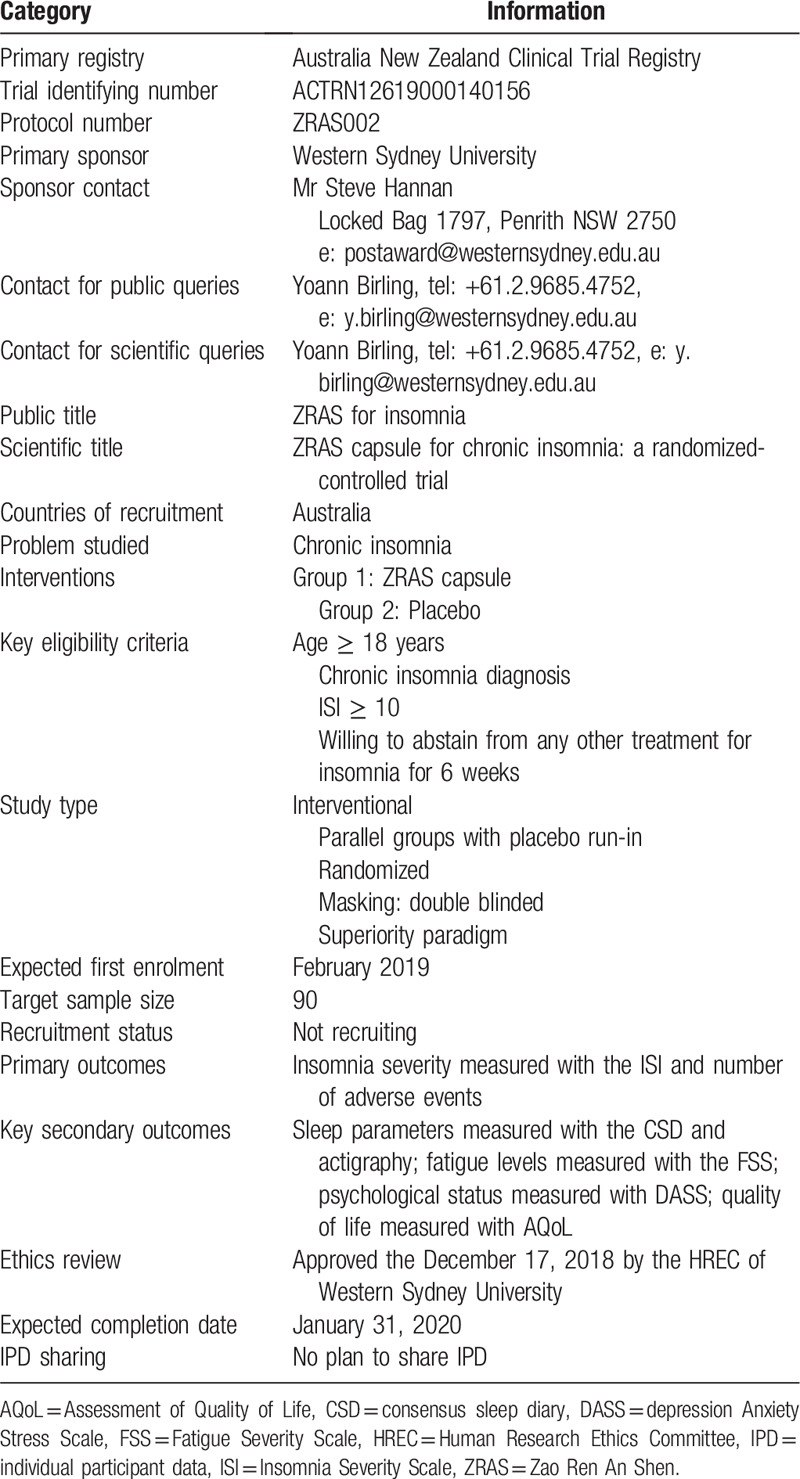
Trial registration data.

### 2.2 Settings

The 3 visits of the trial will be conducted at 4 campuses of Western Sydney University, that is, Parramatta City campus, Bankstown Campus, Westmead campus, and Campbelltown campus around Sydney, Australia.

### 2.3 Participants

#### 2.3.1 Recruitment strategy

Participants will be recruited through advertisement and referrals from the February 15, 2019 to the November 29, 2019. Ads will be put in social media (e.g., Facebook and Wechat) and poster and flyers in public places (e.g., Universities, libraries), pharmacies and healthcare practices. Pharmacists and general practitioners around the trial sites will be contacted for potential referrals.

#### 2.3.2 Screening procedures

The potential participant will be contacted by phone for a prescreening assessment before the first visit. During the first visit, a medical interview including the Mini International Neuropsychiatric Interview and the sleep disorders section of the Structured Clinical Interview for the Diagnostic and Statistical Manual of Mental Disorders, 5th edition (DSM-5) will be conducted by the principal investigator (YB), who is a Chinese medicine practitioner trained in mental disorder diagnosis. Between the first and the second visit, the participant will undergo blood tests including full blood count (FBC), liver function test (LFT), and electrolytes-urea-creatinine (EUC) at Laverty Pathology, if needed, and an oximetry assessment at home using the WristOx (Nonin) if sleep apnea is suspected. A general practitioner involved in the trial (NA) will crosscheck the medical eligibility of the participant. Enrolment will be confirmed during the third visit, after a re-assessment of insomnia severity.

#### 2.3.3 Eligibility criteria

The eligibility criteria, based on the diagnosis criteria for insomnia disorder (307.42) from the DSM-5,^[1]^ are as following:

Inclusion criteria:

1. 18 years old or older.
2. Complaint of difficulty initiating or maintaining sleep occurring at least 3 times per week for at least 3 months despite adequate opportunity to sleep, and resulting in distress or impaired daytime functioning.
3. ISI score ≥ 10.
4. Willing to abstain from any other treatment for insomnia, including pharmaceutical treatment, CAM, and psychotherapy, during the baseline and intervention periods.
5. Willing to use birth control methods and to not donate sperm during the baseline and intervention periods, or sterile.
6. Ability to understand and speak English.

Exclusion criteria:

1. Imminent need of psychiatric (e.g., suicide risk) or medical care (e.g., stroke).
2. Abnormal blood tests, including FBC, LFT, and electrolytes-urea-creatinine, within the last 6 months, if not approved by a general practitioner.
3. No evidence that substance use (e.g., caffeine or a medication), coexisting medical conditions or mental disorders, including sleep–wake disorder, do not adequately explain the predominant complaint of insomnia.
4. Use of any other treatment for insomnia less than 14 days prior to randomization.
5. Any psychotic disorder or bipolar disorder if not appropriately treated or stable for <2 years.
6. Alcohol or drug addiction.
7. Other mental disorders such as major depressive disorder or generalized anxiety disorder, if the disorder is untreated or treated for less than one month.
8. Cognitive impairment preventing the participant to understand the trial instructions, complete questionnaires or provide informed consent.
9. Allergy history to any of the ingredient of the ZRAS capsule or the placebo.
10. Taking a Warfarin-type anticoagulant,^[58,59]^ quetiapine, clozapine, or olanzapine.^[60]^
11. Women being pregnant or breast feeding.
12. Considered not suitable for the trial by the investigator.

### 2.4 Randomization and blinding

A randomization sequence without stratification will be generated using computer-generated random numbers by a NICM Heath Research institute researcher not directly affiliated with this study to ensure blinding. The sequence generated will be used to identify the investigational product. The participants will be randomized during the second visit of the trial. Both the investigator and the participants will be blinded at recruitment and during the intervention. The codes will be broken in case of emergency, such as a serious adverse event that requires knowledge of the treatment being taken in order to manage a participant's condition.

### 2.5 Interventions

The investigational product is ZRAS capsule, a CHM product composed of Suan zao ren (Ziziphi Spinosae Semen, 417 mg/capsule), Wu Wei Zi (Schisandrae Chinensis Fructus, 70 mg/capsule), and Dan Shen (Salviae Miltiorrhizae Radix et Rhizoma, 167 mg/capsule). The placebo, composed of microcrystalline cellulose, calcium hydrogen phosphate dehydrate, carob pod powder, silicon dioxide, and magnesium stearate, matches the active in terms of appearance and taste. The participant will be asked to take 3 capsules of placebo (2.16 g in total) orally once a day one hour before bedtime for one week. After randomization, the participant will be asked to take 3 capsules of ZRAS (2.28 g in total) or placebo (2.16 g in total) orally once a day one hour before bedtime for 4 weeks. Both ZRAS capsule and the placebo were provided by Global Therapeutics, Pty, Ltd. The participant will be asked to refrain from taking any other treatment for insomnia during the week preceding and the 5 weeks following the first visit, except for rescue medication.

### 2.6 Assessments

#### 2.6.1 Primary outcomes

##### 2.6.1.1 Insomnia severity

Insomnia severity will be measured with the Insomnia Severity Index (ISI). The ISI ^[61]^ is a brief 7-item scale used to assess perceived insomnia severity and consequences in accordance with the diagnostic criteria for insomnia of the Diagnostic and Statistical manual of Mental Disorders, 4th edition, and the International Classification of Sleep Disorders. Unlike the Pittsburgh Sleep Quality Index, another questionnaire commonly used for insomnia assessment, the ISI is specific to insomnia disorder. The ISI has good psychometric properties ^[61]^ and can be used to detect insomnia cases and evaluate treatment response.^[62]^ The range of the scale is 0 to 28 points and the score is positively associated with insomnia severity.

##### 2.6.1.2 Adverse events

The participants will be encouraged to report any AE at any time during the intervention or follow-up period. A description of the AE will be recorded along with the seriousness, severity, relationship with the investigational product and expectedness of the AE in the AE form. The number of adverse events will be used as the primary outcome for safety. Serious adverse events will be reported to the Western Sydney University Human Research Ethics Committee and the Therapeutics Goods Administration. All significant safety issues and suspected unexpected serious adverse reactions will be reported within 72 hours to the trial site (Western Sydney University).

#### 2.6.2 Secondary outcomes

##### 2.6.2.1 Questionnaires

The Depression Anxiety Stress Scale 21-item ^[63]^ is a self-report questionnaire composed of 21 items assessing the levels of depression, anxiety, and stress of the subject. The Assessment of Quality of Life ^[64]^ questionnaire is a self-report instrument used to assess health-related quality of life. This study will use the 4-dimension version of the questionnaire, which measures independence, relationships, mental status, and senses. The Fatigue Severity Scale ^[65]^ is a 7-point Likert scale that measures different aspects of fatigue. The questionnaires used in this study have all acceptable psychometric properties.^[64,66,67]^

##### 2.6.2.2 Sleep parameters

Sleep parameters, including total sleep time (TST), sleep onset latency, number of awakenings, wake after sleep onset duration, and sleep efficiency (i.e., the ratio between TST and time in bed), will be recorded with the Consensus Sleep Diary (CSD) and an actigraph. The CSD is a self-report table in which the participant reports his sleep-related behaviors, including time and duration.^[68]^ The CSD has been validated in an insomnia disorder population. An actigraph is a device, usually wrist worn, capable of measuring gross motor activity. Sleep parameters can be calculated by software using the 24-hour gross motor activity measured by the actigraph. In this study, we will use the readiband (Fatigue Science, Canada), which has been validated against polysomnography.^[69]^ The sleep parameters will be averaged on one week if at least 4 days of data are available.

##### 2.6.2.3 Expectancy, adherence and acceptability

Outcome expectancy will be measured with the Credibility and Expectancy Questionnaire (CEQ). This questionnaire was developed in 1972 to assess the credibility of placebo behavioral interventions.^[70]^ The revised version of the questionnaire has acceptable psychometric properties ^[71,72]^ and has been widely used to assess expectancy in clinical trials. The count of the unused capsules collected during the third visit will serve as estimation for adherence, which will be also monitored with the CSD. The third item of the CEQ will be used to assess acceptability.

##### 2.6.2.4 Baseline assessments

Besides the outcome measurements, life events and night pain levels will be collected at baseline in order to assess the influence of these measures on the outcomes. Life events will be assessed with the List of Threatening Events (LTE), a list of 12 life-event categories with considerable long-term contextual threat.^[73]^ The LTE has acceptable psychometric properties.^[74]^ Night pain levels will be assessed with a 100° pain visual analog scale (VAS), adapted for the circumstances with the question: “what is your average pain level when you are trying to sleep?” The VAS is a valid and sensitive instrument to assess pain levels.^[75,76]^

##### 2.6.2.5 Chinese Medicine diagnosis

A Chinese Medicine (CM) diagnosis (also known as “pattern differentiation”) will be determined on the basis of a self-report questionnaire and an examination of the complexion, the pulse and the tongue by the principal investigator (YB). The items of the questionnaire and the examination are based on a widely recognized CM manual.^[77]^ Each positive symptoms or sign counts for 1 point and the participant is diagnosed with the pattern that has the highest score, or the pattern determined by the principal investigator in case of equal score. The CM diagnosis will be used as an efficacy outcome and a predictor variable for efficacy and safety outcomes.

### 2.7 Clinical significance

A decrease of 7 points in the ISI score was found to be an indicator of moderate improvement for insomnia disorder.^[62]^ Therefore, a decrease of 7 points or more in the ISI score at post-treatment compared to pre-treatment will be considered clinically significant.

### 2.8 Data collection and management

Electronic data will be collected with Research Electronic Data Capture (REDCap) database. REDCap surveys utilize a secure connection to the server for data collection which does not record IP addresses of participants. The questionnaires will be completed by the participant on REDCap via an iPad during the 3 face-to-face visits and by the investigator during the mid-treatment and follow-up phone interviews according to the participant's oral answers. A secured link to the database for the CSD will be sent every day via email to the participant. Paper forms for the sleep diary will be offered as an option for those who prefer them. For the follow-up, these will be supplied along with reply paid envelopes. Electronic data will be stored in REDCap for the duration of the trial and paper data will be stored in a locked cabinet at the trial site. Personal information will be kept in REDCap and an excel file protected with a password before and during the trial, and in a masterlist stored in a shared drive with restricted access after the trial. The final trial dataset will be accessible only to the investigators. In order to avoid missing data, the importance of complete data collection will be emphasized at enrolment and participants who withdraw from the study will be asked to complete the data collection, if willing. The research team will monitor data on an ongoing basis.

### 2.9 Statistical analysis

Continuous data will be analyzed with linear mixed effects model, the data being corrected for baseline characteristics; mean, standard deviation, effect size, 95% CI and *P*-value will be reported. Dichotomous data will be analyzed with Chi-square and counts, percentages, and *P*-value will be reported. We will analyze the final vale of the outcomes with a significance level of α=0.05. Both an intention-to-treat analysis and a per-protocol analysis will be conducted, the intention-to-treat analysis being of primary interest as it reflects better the effectiveness of the investigational product. The effect of expectancy and CM diagnosis on the trial outcomes will also be analyzed. The statistical analysis will be performed with SPSS 25 (IBM).

### 2.10 Sample size

The standard deviation of the ISI total score in an insomnia population is 4.1.^[61]^ A difference of 7 points between baseline and post-treatment is considered as clinically significant.^[62]^ As placebo would give a decrease of approximately 4 points,^[78]^ the difference between the 2 groups at post-treatment has to be of 0.73 standard deviations. Using a 0.05 significance level, 90% power and allowing for 10% dropout rate, a sample size of 90 participants, 45 in each group, will be required.

### 2.11 Ethics and dissemination

Informed consent will be obtained by the principal investigator (YB) from all the participants during the first visit of the trial. The participant will be withdrawn from the study in case a serious AE or the worsening of the condition justifies the withdrawal, and the participant will be allowed to withdraw at any time for any reason. The trial will be conducted in accordance with the World Medical Association Declaration of Helsinki and the National Statement on Ethical Conduct in Human Research. Ethic approval has been obtained from the Human Research Ethic Committee (HREC) of Western Sydney University (ethics approval number H12990). Any protocol revision will be implemented after HREC approval only. The trial results and the allocation will be disseminated to the participant after the end of the trial and the trial result will be reported in a peer-reviewed journal.

### 2.12 Trial monitoring

This trial, which is part of YB doctoral thesis project, will be monitored by XZ, AB, and JS, the supervisors of YB. Monthly meetings will be held to discuss trial progression and monitor data collection. NA will monitor the trial in terms of AEs management. A meeting can be called at any time by any member of the research team in the event of participant risk concerns in the trial. Based on this information, the research team will stop the trial if it becomes possible that risk might outweigh benefit for the participants as a group. Based on existing safety information of the investigational products, this scenario is regarded as highly unlikely.

## 3 Discussion

The objective of this study is to assess if ZRAS is relatively safe and effective for chronic insomnia compared to placebo. This study, which has a stringent design with randomization and double blinding, will provide high-quality evidence for or against the use of ZRAS capsule as an alternative treatment for chronic insomnia.

If the hypothesis is verified (i.e., ZRAS is relatively safe and more effective than placebo), ZRAS may be recommended if the current recommended treatments (i.e., pharmacotherapy and CBT-I) are ineffective, not available or if the patient refuses these treatments. More RCTs comparing ZRAS capsule to pharmacotherapy or CBT-I would be also needed to assess if ZRAS should be used as a first line or alternative treatment. RCTs comparing ZRAS capsule in addition to recommended treatments to recommended treatments alone would help us to understand the value of adding ZRAS capsule to these treatments.

The strengths of this study are:

- The use of a third-party randomization scheme with allocation concealment and double blinding.
- The use of a placebo similar in appearance and taste to the active.
- The combination of subjective (i.e., with the CSD) and objective (i.e., with actigraphy) measures of sleep.
- A comprehensive assessment encompassing fatigue, psychological status, quality of life, satisfaction and acceptability.
- The use of a CM diagnosis.

## Acknowledgments

The authors would like to thank Yang Lan for her work about the trial equipment and the investigator's brochure, Suzannah Bourchier for her support on technical and regulatory matters, Paul Fahey for his help about statistical matters, Danielle Parker, Margaret He, Christine Murray, Dianna Porter, Mingxian Jia, Kylie Barr, and Sara Low for their comments and help on the study protocol, Natalie Connor, Jennifer Rodda, Beatrice Venkataya and Mike Armour for their help regarding the recruitment strategy. YB is a recipient of a Blackmores-NICM scholarship. JS is supported by an NHMRC Clinical Research Fellowship (APP1125000).

## Author contributions

Yoann Birling drafted the present manuscript and Xiaoshu Zhu, Alan Bensoussan, Jerome Sarris, Nicole Avard reviewed and edited the manuscript.

**Conceptualization:** Yoann Birling.

**Supervision:** Alan Bensoussan, Jerome Sarris, Xiaoshu Zhu.

**Writing – original draft:** Yoann Birling.

**Writing – review & editing:** Alan Bensoussan, Jerome Sarris, Nicole Avard, Xiaoshu Zhu.

Yoann Birling orcid: 0000-0003-1478-6705.

## References

[1] American Psychiatric Association. Diagnostic and Statistical Manual of Mental Disorders (DSM-5). Arlington, VA: American Psychiatric Association; 2013.

[2] American Academy of Sleep Medicine. International Classification of Sleep Disorders. 3rd edn. Darien, IL: American Academy of Sleep Medicine; 2014.

[3] Benbir G Demir AU Aksu M Prevalence of insomnia and its clinical correlates in a general population in Turkey. Psychiatry Clin Neurosci 2015;69:543–52. 25384688 10.1111/pcn.12252

[4] Castro LS Poyares D Leger D Objective prevalence of insomnia in the São Paulo, Brazil epidemiologic sleep study. Ann Neurol 2013;74:537–46. 23720241 10.1002/ana.23945

[5] Uhlig BL Sand T Ødegård SS Prevalence and associated factors of DSM-V insomnia in Norway: the Nord-Trøndelag Health Study (HUNT 3). Sleep Med 2014;15:708–13. 24767721 10.1016/j.sleep.2014.01.018

[6] Baglioni C Battagliese G Feige B Insomnia as a predictor of depression: a meta-analytic evaluation of longitudinal epidemiological studies. J Affective Disorders 2011;135:10–9. 10.1016/j.jad.2011.01.011 21300408

[7] Neckelmann D Mykletun A Dahl AA Chronic insomnia as a risk factor for developing anxiety and depression. Sleep 2007;30:873–80. 17682658 10.1093/sleep/30.7.873 PMC1978360

[8] Affleck G Urrows S Tennen H Sequential daily relations of sleep, pain intensity, and attention to pain among women with fibromyalgia. Pain 1996;68:363–8. 9121825 10.1016/s0304-3959(96)03226-5

[9] Suka M Yoshida K Sugimori H Persistent insomnia is a predictor of hypertension in Japanese male workers. J Occup Health 2003;45:344. 14676413 10.1539/joh.45.344

[10] Phillips B Mannino DM Do insomnia complaints cause hypertension or cardiovascular disease? J Clin Sleep Med 2007;3:489. 17803012 PMC1978336

[11] Scalo J Desai P Rascati K Insomnia, hypnotic use, and health-related quality of life in a nationally representative sample. Qual Life Res 2015;24:1223–33. 25432884 10.1007/s11136-014-0842-1

[12] Parthasarathy S Vasquez MM Halonen M Persistent insomnia is associated with mortality risk. Am J Med 2015;128:268.e262–75.e262. 25447616 10.1016/j.amjmed.2014.10.015 PMC4340773

[13] Daley M Morin CM LeBlanc M The economic burden of insomnia: direct and indirect costs for individuals with insomnia syndrome, insomnia symptoms, and good sleepers. Sleep 2009;32:55–64. 19189779 PMC2625324

[14] Morgenthaler T Kramer M Alessi C Practice parameters for the psychological and behavioral treatment of insomnia: an update. An American Academy of Sleep Medicine Report. Sleep 2006;29:1415. 17162987

[15] Schutte-Rodin S Broch L Buysse D Clinical guideline for the evaluation and management of chronic insomnia in adults. J Clin Sleep Med 2008;4:487–504. 18853708 PMC2576317

[16] Qaseem A Kansagara D Forciea MA Management of chronic insomnia disorder in adults: a clinical practice guideline from the American College of Physicians. Ann Int Med 2016;165:125–33. 27136449 10.7326/M15-2175

[17] Ree M Junge M Cunnington D Australasian Sleep Association position statement regarding the use of psychological/behavioral treatments in the management of insomnia in adults. Sleep Med 2017;36:S43–7. 28648226 10.1016/j.sleep.2017.03.017

[18] Bertisch SM Herzig SJ Winkelman JW National use of prescription medications for insomnia: NHANES 1999–2010. Sleep 2014;37:343–9. 24497662 10.5665/sleep.3410 PMC3900622

[19] Walsh JK Roth T Kryger MH Roth T Dement WC Pharmacologic treatment of insomnia: benzodiazepine receptor agonists. Principles and Practices of Sleep Medicine 5th edn Glendenning, Australia: Saunders; 2011 905–15.

[20] Buysse DJ Insomnia. JAMA 2013;309:706–16. 23423416 10.1001/jama.2013.193 PMC3632369

[21] Licata SC Rowlett JK Abuse and dependence liability of benzodiazepine-type drugs: GABA A receptor modulation and beyond. Pharmacol Biochem Behav 2008;90:74–89. 18295321 10.1016/j.pbb.2008.01.001 PMC2453238

[22] Hajak G Müller W Wittchen H-U Abuse and dependence potential for the non-benzodiazepine hypnotics zolpidem and zopiclone: a review of case reports and epidemiological data. Addiction 2003;98:1371–8. 14519173 10.1046/j.1360-0443.2003.00491.x

[23] Morin CM Gaulier B Barry T Patients’ acceptance of psychological and pharmacological therapies for insomnia. Sleep 1992;15:302. 1519003 10.1093/sleep/15.4.302

[24] Vincent N Lionberg C Treatment preference and patient satisfaction in chronic insomnia. Sleep 2001;24:411. 11403525 10.1093/sleep/24.4.411

[25] Edinger JD Sampson WS A primary care “friendly” cognitive behavioral insomnia therapy. Sleep 2003;26:177–82. 12683477 10.1093/sleep/26.2.177

[26] Morin CM LeBlanc M Bélanger L Prevalence of insomnia and its treatment in Canada. Can J Psychiatry 2011;56:540–8. 21959029 10.1177/070674371105600905

[27] Bertisch SM Wells RE Smith MT Use of relaxation techniques and complementary and alternative medicine by American adults with insomnia symptoms: results from a national survey. J Clin Sleep Med 2012;8:681. 23243402 10.5664/jcsm.2264 PMC3501665

[28] Pearson NJ Johnson LL Nahin RL Insomnia, trouble sleeping, and complementary and alternative medicine: Analysis of the 2002 national health interview survey data. Arch Int Med 2006;166:1775–82. 16983058 10.1001/archinte.166.16.1775

[29] Lee KH Tsai YT Lai JN Concurrent use of hypnotic drugs and Chinese herbal medicine therapies among Taiwanese adults with insomnia symptoms: a population-based study. Evid Based Complement Alternat Med 2013;2013:987862. 24204397 10.1155/2013/987862 PMC3800591

[30] Yeung WF Chung KF Yung KP The use of conventional and complementary therapies for insomnia among Hong Kong Chinese: A telephone survey. Complement Ther Med 2014;22:894–902. 25440381 10.1016/j.ctim.2014.08.001

[31] Frass M Strassl RP Friehs H Use and acceptance of complementary and alternative medicine among the general population and medical personnel: a systematic review. Ochsner J 2012;12:45. 22438782 PMC3307506

[32] Ni XJ Shergis JL Guo XF Updated clinical evidence of Chinese Herbal Medicine for insomnia: a systematic review and meta-analysis of randomized controlled trials. Sleep Med 2015;16:1462–81. 26611943 10.1016/j.sleep.2015.08.012

[33] Yeung WF Chung KF Poon MMK Chinese Herbal Medicine for insomnia: a systematic review of randomized controlled trials. Sleep Med Rev 2012;16:497–507. 22440393 10.1016/j.smrv.2011.12.005

[34] Zhang Y Qi Y Wu Y Dose–effect and time–effect relationship of improving sleep effect of Zao Ren An Shen granules and its influence on mice brain cell factors. China Pharm 2015;24:32–4.

[35] Zhang Y Wu Y Qi Y The mechanism study on the hypnotic effect of Zao Ren An Shen granule. Chin Tradit Patent Med 2016;38:2268–70.

[36] Shergis JL Ni X Sarris J Ziziphus spinosa seeds for insomnia: a review of chemistry and psychopharmacology. Phytomedicine 2017;34:38–43. 28899507 10.1016/j.phymed.2017.07.004

[37] Monti JM Pandi-Perumal SR Jacobs BL Serotonin and Sleep: Molecular, Functional and Clinical Aspects. Berlin, Germany: Springer Science & Business Media; 2008.

[38] Fang XS Hao J Zhou H Pharmacological studies on the sedative-hypnotic effect of Semen Ziziphi spinosae (Suanzaoren) and Radix et Rhizoma Salviae miltiorrhizae (Danshen) extracts and the synergistic effect of their combinations. Phytomedicine 2010;17:75–80. 19682877 10.1016/j.phymed.2009.07.004

[39] Wang L Zhang M Yan C Study on acute toxicity of alcohol-soluble extract of semen ziziphi spinosae. Lishizhen Med Materia Med Res 2009;20:1610–1.

[40] Hou Y Bai W Liu J Study of toxicity and genotoxicity of Dan Shen injections single drug administration. Northwest Pharm J 2017;32:486–9.

[41] Hancke JL Burgos RA Ahumada F Schisandra chinensis (Turcz.) Baill. Fitoterapia 1999;70:451–71.

[42] Chinese Pharmacopoeia Committee. *Pharmacopoeia of the People's Republic of China* Vol 1 2015 ed Beijing, China: China Medical Science Press; 2015.

[43] Qin G Jin H Lu G Controlled observation of Zao Ren An Shen capsule and Clonazepam for the treatment of insomnia. Paper presented at: 2007 Annual Conference of Zhejiang's Psychiatry2007; Lishui, China.

[44] Ren Y Ni F Analysis of the efficacy of Zao Ren An Shen capsule for the treatment of insomnia in older adults. J Guiyang Coll Tradit Chin Med 2007;29:23–4.

[45] Zhang W Shi Z Sun Y Treatment of 32 cases of double-deficiency-of-heart-and-spleen-type insomnia with Zao Ren An Shen capsule. China Pharm 2007 58.

[46] Liu Y Nan D Clinical observation of Zao Ren An Shen capsule for psychophysiological insomnia. Chin J Chin Mater Med 2009;34:1730–1.

[47] Tian G Qin G Study on the efficacy and hemorrheology of Zao Ren An Shen capsule as a treatment of insomnia in older adults. Paper presented at: The 6th Annual Conference of the Chinese Sleep Research Society2010; Chengdu, China.

[48] Xu C Zao Ren An Shen capsule and alprazolam as a treatment for insomnia: a controlled study. Nei Mongol J Tradit Chin Med 2011;30:3.

[49] Li G Gong X Observation of the efficacy of Zao Ren An Shen capsule as a treatment of 30 cases of insomnia. Guiding J Tradit Chin Med Pharmacol 2012;18:53–4.

[50] Huang H Observation of the efficacy of Zao Ren An Shen capsule for the treatment of insomnia. China Health Care Nutr 2013 387–8.

[51] Chen Y Li S Yang L 60 insomnia cases treated with Zao Ren An Shen granule: a clinical study. Hebei J Tradit Chin Med 2014;36:1145–7.

[52] Qin G Tian G Gan J Impact on insomnia patients using polysomnography: a controlled study. Chinese Rural Health Service Admin 2015;35:796–8.

[53] Liang Y Clinical observation of Zao Ren An Shen capsule as a treatment of insomnia in older adults. Med Inform 2016 80–1.

[54] Zhang J The clinical effect of Zao Ren An Shen capsule and Estazolam as a treatment for insomnia patients. Chin J Med Device 2016;29:113.

[55] Liu H Chen F Study on the efficacy of Zao Ren An Shen capsule for the treatment of insomnia in older adults. World Latest Med Inform 2017;17:71.

[56] Wang J Shen W Yao X Analysis of the clinical value of the sleep-improving effect of Zao Ren An Shen capsule. Asia-Pacific Tradit Med 2017;13:137–8.

[57] Wang X Guo C Ma J Analysis of the efficacy and adverse reactions of Zao Ren An Shen capsule and Estazolam for sleep disorders. World Clin Med 2017 102.

[58] Izzat MB Yim APC El-Zufari MH A taste of Chinese medicine!. Ann Thorac Surg 1998;66:941–2. 9768962 10.1016/s0003-4975(98)00624-9

[59] Yu CM Chan JCN Sanderson JE Chinese herbs and warfarin potentiation by ‘Danshen’. J Int Med 1997;241:337–9. 10.1046/j.1365-2796.1997.134137000.x 9159606

[60] Zhang-Jin Z Yao T Xue-Yi W An epidemiological study of concomitant use of chinese medicine and antipsychotics in schizophrenic patients: implication for herb-drug interaction. PLoS One 2011;6:e17239. 21359185 10.1371/journal.pone.0017239 PMC3040227

[61] Bastien CH Vallières A Morin CM Validation of the Insomnia Severity Index as an outcome measure for insomnia research. Sleep Med 2001;2:297–307. 11438246 10.1016/s1389-9457(00)00065-4

[62] Morin CM Belleville G Bélanger L The insomnia severity index: psychometric indicators to detect insomnia cases and evaluate treatment response. Sleep 2011;34:601–8. 21532953 10.1093/sleep/34.5.601 PMC3079939

[63] Lovibond PF Lovibond SH The structure of negative emotional states: Comparison of the Depression Anxiety Stress Scales (DASS) with the Beck Depression and Anxiety Inventories. Behav Res Ther 1995;33:335–43. 7726811 10.1016/0005-7967(94)00075-u

[64] Hawthorne G Richardson J Osborne R The Assessment of Quality of Life (AQoL) instrument: a psychometric measure of Health-Related Quality of Life. Qual Life Res 1999;8:209–24. 10472152 10.1023/a:1008815005736

[65] Krupp LB Larocca NG Muir-Nash J The Fatigue Severity Scale: application to patients with multiple sclerosis and systemic lupus erythematosus. Arch Neurol 1989;46:1121–3. 2803071 10.1001/archneur.1989.00520460115022

[66] Henry J Crawford J The short-form version of the Depression Anxiety Stress Scales (DASS-21): construct validity and normative data in a large non-clinical sample. Brit J Clin Psychol 2005;44:227–39. 16004657 10.1348/014466505X29657

[67] Rosa K Fu M Gilles L Validation of the Fatigue Severity Scale in chronic hepatitis C. Health Qual Life Outcomes 2014;12:90. 24915781 10.1186/1477-7525-12-90 PMC4094687

[68] Carney CE Buysse DJ Ancoli-Israel S The consensus sleep diary: standardizing prospective sleep self-monitoring. Sleep 2012;35:287–302. 22294820 10.5665/sleep.1642 PMC3250369

[69] Russell C Caldwell J Arand D Validation of the fatigue science readiband actigraph and associated sleep/wake classification algorithms. Arch LLC 2000;http://www.fatiguescience.com/wp-content/uploads/2016/09/Readiband-Validation-Accuracy.pdf

[70] Borkovec TD Nau SD Credibility of analouge therapy rationales. J Behav Ther Exp Psychiatry 1972;3:257–60.

[71] Devilly GJ Borkovec TD Psychometric properties of the credibility/expectancy questionnaire. J Behav Ther Exp Psychiatry 2000;31:73–86. 11132119 10.1016/s0005-7916(00)00012-4

[72] Mertens VC Moser A Verbunt J Content validity of the credibility and expectancy questionnaire in a pain rehabilitation setting. Pain Pract 2017;17:902–13. 27911035 10.1111/papr.12543

[73] Brugha T Bebbington P Tennant C The List of Threatening Experiences: a subset of 12 life event categories with considerable long-term contextual threat. Psychol Med 1985;15:189–94. 3991833 10.1017/s003329170002105x

[74] Motrico E Moreno-Küstner B de Dios Luna J Psychometric properties of the List of Threatening Experiences—LTE and its association with psychosocial factors and mental disorders according to different scoring methods. J Affect Disord 2013;150:931–40. 23726778 10.1016/j.jad.2013.05.017

[75] Breivik KE Björnsson AG Skovlund AE A comparison of pain rating scales by sampling from clinical trial data. Clin J Pain 2000;16:22–8. 10741815 10.1097/00002508-200003000-00005

[76] Dworkin HR Turk CD Farrar TJ Core outcome measures for chronic pain clinical trials: IMMPACT recommendations. Pain 2005;113:9–19. 15621359 10.1016/j.pain.2004.09.012

[77] Zhang BL Wu MH Internal Medicine of Chinese Medicine. 4th edn Beijing, China: China Press of Traditional Chinese Medicine; 2017.

[78] Walsh JK Krystal AD Amato DA Nightly treatment of primary insomnia with eszopiclone for six months: effect on sleep, quality of life, and work limitations. Sleep 2007;30:959. 17702264 10.1093/sleep/30.8.959 PMC1978384

